# Seroprevalence of COVID-19 antibodies in the cleaning and oncological staff of a municipal clinic

**DOI:** 10.3205/dgkh000353

**Published:** 2020-07-23

**Authors:** Jörg Epstude, Igor Alexander Harsch

**Affiliations:** 1 Department of Hospital Hygiene, Thuringia Clinic “Georgius Agricola”, Saalfeld/Saale, Germany; 2 Department of Internal Medicine II, Thuringia Clinic “Georgius Agricola”, Saalfeld/Saale, Germany

**Keywords:** antibodies, COVID-19, SARS-CoV-2, serology, cleaning staff, cleaning personnel, hospital hygiene, oncology, cancer

## Abstract

**Aim:** To prevent shedding of the novel COVID-19 virus in hospitals, strict hygiene measures and surveillance of the staff and patients is mandatory. Studying the available literature, we assumed that monitoring of the cleaning staff may sometimes be a “blind spot” in surveillance. Although the cleaning personnel is not entrusted with the medical and nursing care of patients, the extent of patient contacts in this group may be comparable to medical personnel and even increase in times of a visit ban in many hospitals. The aim of this study was to investigate the prevalence of COVID-19 infections already undergone in this group.

**Methods:** Antibody titers (IgA and IgG) against COVID-19 were measured in the cleaning staff from June 15^th^ to 30^th^, 2020 in our clinic. Antibodies against COVID-19 were determined using ELISA (EUROIMMUN™, PerkinElmer, Inc. Company). For purposes of comparison, the same procedure was performed in the staff of the oncology ward, who were regarded as an important group due to their high-risk patients.

**Results:** During the study period, 45 members of the cleaning staff and 20 members of the oncology ward were tested. Significantly elevated IgA antibody titers were detected in 1 person in the first group and in 1 person in the second group. Significantly elevated IgG antibody titers were not detected in the first group and in 1 person of the second group. In case of positive or indeterminate testing, swabs for direct virus detection were taken, but were negative in all cases.

**Conclusion:** The prevalence of already undergone infections in both groups is low, as to be expected due to the still low incidence of COVID-19 infections in the German federal state of Thuringia. However, the presence of such antibodies in the cleaning personnel demonstrates the need for equally strict surveillance in this group.

## Zusammenfassung

**Einleitung:** Um die Verbreitung des neuartigen COVID-19-Virus in Krankenhäusern zu verhindern, sind strenge Hygienemaßnahmen und die Überwachung des Personals und der Patienten obligatorisch. Nach Sichtung der verfügbaren Literatur entsteht der Eindruck, dass die Überwachung des Reinigungspersonals manchmal ein „blinder Fleck“ bei der Überwachung ist. Obwohl das Reinigungspersonal nicht mit der medizinischen und pflegerischen Versorgung der Patienten betraut ist, kann das Ausmaß der Patientenkontakte in dieser Gruppe mit denen der Pflege vergleichbar sein und in Zeiten eines Besuchsverbots in vielen Krankenhäusern sogar zunehmen. Ziel dieser Studie war es, die Prävalenz bereits überstandener COVID-19-Infektionen in dieser Gruppe zu untersuchen.

**Methoden:** Die Antikörpertiter (IgA und IgG) gegen COVID-19 wurden beim Raumpflegepersonal vom 15. bis 30. Juni 2020 in unserer Klinik bestimmt. Die Antikörperbestimmung gegen COVID-19 wurde mit einem ELISA (EUROIMMUN^TM^, PerkinElmer, Inc. Company) durchgeführt. Zu Vergleichszwecken wurde bei den Mitarbeitern der Onkologiestation das gleiche Procedere vorgenommen, da wir diese Mitarbeiter aufgrund ihrer Hochrisikopatienten als ebenfalls für eine Überwachung wichtige Gruppe betrachteten.

**Ergebnisse:** Während des Studienzeitraums wurden 45 Mitarbeiter des Raumpflegepersonals und 20 Mitglieder der Onkologiestation getestet. Signifikant erhöhte IgA-Antikörpertiter wurden in beiden Gruppen bei je 1 Person nachgewiesen. Signifikant erhöhte IgG-Antikörpertiter wurden in der ersten Gruppe nicht und in der zweiten Gruppe bei 1 Person nachgewiesen. Bei positiven und grenzwertigen Tests wurden Abstriche für einen direkten Virusnachweis entnommen, die jedoch in allen Fällen negativ waren.

**Diskussion:** Die Prävalenz bereits durchgemachter Infektionen ist in beiden Gruppen gering, was aufgrund der noch geringen Inzidenz von COVID-19-Infektionen im Bundesland Thüringen zu erwarten war. Das Vorhandensein solcher Antikörper beim Raumpflegepersonal zeigt jedoch, dass auch in dieser Gruppe eine strikte Überwachung sinnvoll ist.

## Introduction

The novel infection COVID-19 raises concerns about nosocomial infections and severe acute respiratory syndrome coronavirus 2 (SARS-CoV-2) transmission from patients to healthcare workers and vice versa, as well as infection of both groups in everyday life. The Thuringia Clinic Saalfeld is a municipal care hospital with presently 618 beds. In an attempt to evaluate the effectiveness of the hygiene measures taken so far in terms of the COVID-19 pandemic, we examined the COVID-19 antibody titers in two groups of clinic employees.

The first group examined was the cleaning staff working throughout the house, and the second was the medical and nursing staff of the oncology ward. Given limited financial resources, the reason for this selection was that the epidemiological situation is hardly known in the first group. To our surprise, a Medline search (keywords: COVID-19; cleaning staff/cleaning personnel; hospital hygiene) generated results for professional healthcare workers focusing on outbreak situations (e.g., [1], [2]), but not for hospital cleaning personnel. The decision to investigate the oncological staff resulted from the special risk profile of patients on the oncology ward.

The cleaning personnel are not entrusted with the immediate medical and nursing care of the patients, but the extent of patient contacts in this group may be comparable to medical personnel. Furthermore, there is reason to believe that the ban on visits by relatives and acquaintances in many clinics can even lead to an increase in contact between patients and cleaning staff. Obviously, the cleaning personnel in a clinic must also follow the same protective measures as medical personnel when in contact with patients. However, the current trend toward “outsourcing” may result in inadequate communication of information and performance of procedures. Furthermore, these measures may not necessarily be conducted by health professionals or be given to persons who may have limited German-language skills.

As for the oncological staff, it is important to consider the well-established risk factors associated with mortality in COVID-19: age [3], hypertension, coronary heart disease, and diabetes [4]. However, this may also include cancer, since the patients are more susceptible to infection due to their immunosuppressive state caused by the malignancy itself and oncological therapies. Given a COVID-19 infection, this may cause a poorer prognosis [5]. However, although some authors demonstrated that patients with cancer might have a higher risk of COVID-19 than cancer-free individuals and that patients with cancer had poorer COVID-19 outcomes, the number of patients investigated was low (n=18) and the authors suggested a nationwide analysis.

Preventive strategies in hospitalized cancer patients include the use of disposable personal protective equipment for health workers and patients, social distancing in waiting rooms and wards, prohibiting visitors from accompanying patients, and alerting health workers to minimize the time spent in the hospital rooms [6]. In terms of cancer therapy, is important to consider possibly delaying treatment depending on the tumor biology and staging, converting intravenous treatment to an oral regimen where possible, and adopting less toxic chemotherapy to limit complications requiring re-hospitalization [7], [8]. Recommendations of the European Society for Medical Oncology do of course also include the staff of oncological wards (“Protect yourself to protect your patients”) [8]. 

Despite the restrictions in everyday life, the risk for any member of the hospital staff to become infected in the social or home environment outside the clinic remains unaffected. As for the surveillance of any staff, health diaries with a regular documentation of possible symptoms such as fever or cough are an option. Unfortunately, the insidious onset of the COVID-19 infection (that can manifest without any obvious clinical symptoms, such as fever, in the early phase) and the long incubation period (up to 24 days) limits the effectivity [7]. A regular testing by using oropharyngeal smears using a multiplex real-time PCR with specific gene probes is the “gold standard”. Unfortunately, COVID-19 testing may take about a day, is expensive and can be problematic due to limited laboratory capacities. Furthermore the method can have problems in terms of “early” and post negative detection [9]. The value of regular antibody testing is still being discussed [10], and solid data on the extent of active or undergone infections among the staff described are hardly available. Such testing may help in understanding the extent of recently acquired and already overcome infections [11].

Thus, the particular focus of our investigation was to collect data on how many members of the two groups could have already experienced a COVID-19 infection. 

## Methods

### Persons and proceedings

After an information session, obtaining informed consent, and with approval by the Ethics Committee of the State Medical Association of Thuringia, we examined the IgA and IgG antibody titers of the housekeeping staff (n=45) and the oncological staff (n=20) (4 doctors, 16 nurses) in our clinic from June 15^th^ to 30^th^, 2020.

### Antibodies

An enzyme-linked immunosorbent assay (ELISA) is used for determining antibodies against SARS-CoV-2 (EUROIMMUN™, a PerkinElmer, Inc. company). The assay is CE (Conformité Européenne)-certified and IVD (In Vitro Diagnostic)-approved. The specificity of the test for IgG is given as 98.5% by the distributor and as 92.5% for IgA. Validation in our house was done from known cases of undergone COVID-19 infection. Antibody titers below 0.8 were negative and after discussion with the laboratory doctors, we considered titers of 2 and above to be reliable and significant.

### Swab

SARS-CoV-2 is diagnosed using oropharyngeal smears and a multiplex real-time PCR with three specific gene probes (N, E and RdRp). The abbreviations refer to structural proteins of the coronavirus. These are nucleocapsid protein (N), small envelope protein (E) and RNA-dependent RNA polymerase (RdRp). The detection limit is 100 RNA copies/reaction.

## Results

The numerical imbalance between the two groups does not allow statistical comparative testing, which is why the data are presented as a table (Table 1 (Tab. 1)).

In case of elevated antibodies against COVID-19, an oropharyngeal swab for COVID-19 was performed, which was negative in all cases. 

Since March 3^rd^, 2020, when the first case of SARS-CoV pneumonia was detected in our clinic as well as in the German federal state of Thuringia, only one nurse of the oncological staff reported possible symptoms of COVID-19 infection and was tested positive by PCR (but without transmission to the patients). This is also reflected by significantly elevated IgA and IgG titers. As for the cleaning staff, the person with elevated IgA titers was asymptomatic. It is noteworthy that on the oncology ward, none of the cleaning personnel had significantly elevated antibody titers.

## Discussion

According to the website of the Robert Koch Institute, 3,265 cases of COVID-19 infection have been identified in the German federal state of Thuringia [12]. The 7-day incidence is 1 case (accessed July 3^rd^). This means that the incidence of disease is relatively low compared to the total incidence in Germany (195,674 cases as of July 3^rd^, 2020). This may be reflected by the rather low frequency of infections already undergone in both groups examined in our clinic.

However, our still-limited understanding of the kinetics of rise and fall of antibodies in COVID-19 raises the question of whether antibodies have failed to form in some persons or whether they have already decreased below the detection limit.

Examining the timeline of antibody formation and using an ELISA by another distributor, Xiang et al. [11] tested serological IgM and IgG antibodies in 216 serum samples of 85 confirmed COVID-19 pneumonia patients. The IgM and IgG antibodies were detected as positive as early as the 4^th^ day after onset, and the seropositivity rate of IgM increased gradually. However, IgG increased sharply by the 12^th^ day after onset. In an attempt to address not only the kinetics of antibody formation in terms of their rise, but also their decline in serum, Sun et al. [13] analyzed longitudinal blood samples from 38 patients (11 intensive care unit [ICU] patients, 27 non-ICU patients). IgM antibodies against nucleocapsid protein (N) and matrix protein (M) had different kinetics in the two groups and may thus be dependent on the severity of the disease. Those authors reported the dynamic pattern of IgM and IgG against N and M as “chaotic” in ICU patients. Understanding antibody kinetics is still problematic because our information often relies on patients who were hospitalized because of COVID-19 infection and who were symptomatic [14]. Furthermore, the limits of our current knowledge are illustrated by surprising observations that some people (even hospitalized patients) who present positive results from molecular tests do not have detectable levels of protective IgG antibodies or neutralizing antibodies [14]. Such open questions, concerns and limitations have to be kept in mind when analyzing the data of studies performed to analyze the epidemic situation (focusing on already overcome infections).

The current data situation for antibody tests in Germany’s general population shows a very inconsistent picture regarding already overcome infections with COVID-19 [15]. For example, the analysis of IgA and IgG levels in the “Heinsberg” study (919 study participants living in 405 households) measured in plasma samples of all study participants by ELISA (EUROIMMUN™) showed 18.5% of the study participants to be IgA positive and 13.6% IgG positive. Contrastingly, in a study in blood donors in Hamburg from April 6 to 10, only one previously unknown SARS-CoV-2 infection was detected serologically (0.3%) in 300 persons; from May 4 to 6, there were two previously unknown cases of SARS-CoV-2 infection in 288 blood donors (0.7%), and from June 2 to 5 with 326 blood donors, again only one previously unknown SARS-CoV-2 infection (0.3%) [16]. 

In principle, an analysis of COVID-19 antibody titers would be desirable for evaluating the infection status of the entire hospital staff and – combined with molecular testing to detect the most recent infections – obtaining a “complete” epidemiological picture. Our limitation to two groups of people resulted from financial constraints; the selection criteria were justified and described above. Due to the present lack of data, it may also be speculated that the cleaning staff is a blind spot in surveillance. Selecting the oncological staff as the comparison group was based on the particular risk situation of the patients. For similar reasons, areas such as dialysis, neonatal (ICU) stations and transplantation wards would also be of interest. 

## Conclusion

Our results for the cleaning staff show that antibodies against COVID-19 are detectable, as expected. This emphasizes the need for strict surveillance of this group in hospitals.

## Notes

### Competing interests

The authors declare that they have no competing interests.

### Funding

There was no financial support.

### Acknowledgements

We thank the individuals who provided blood samples to support scientific research and I. Nichterlein and S. Werschowitz for the organisatorial support.

## Figures and Tables

**Table 1:**
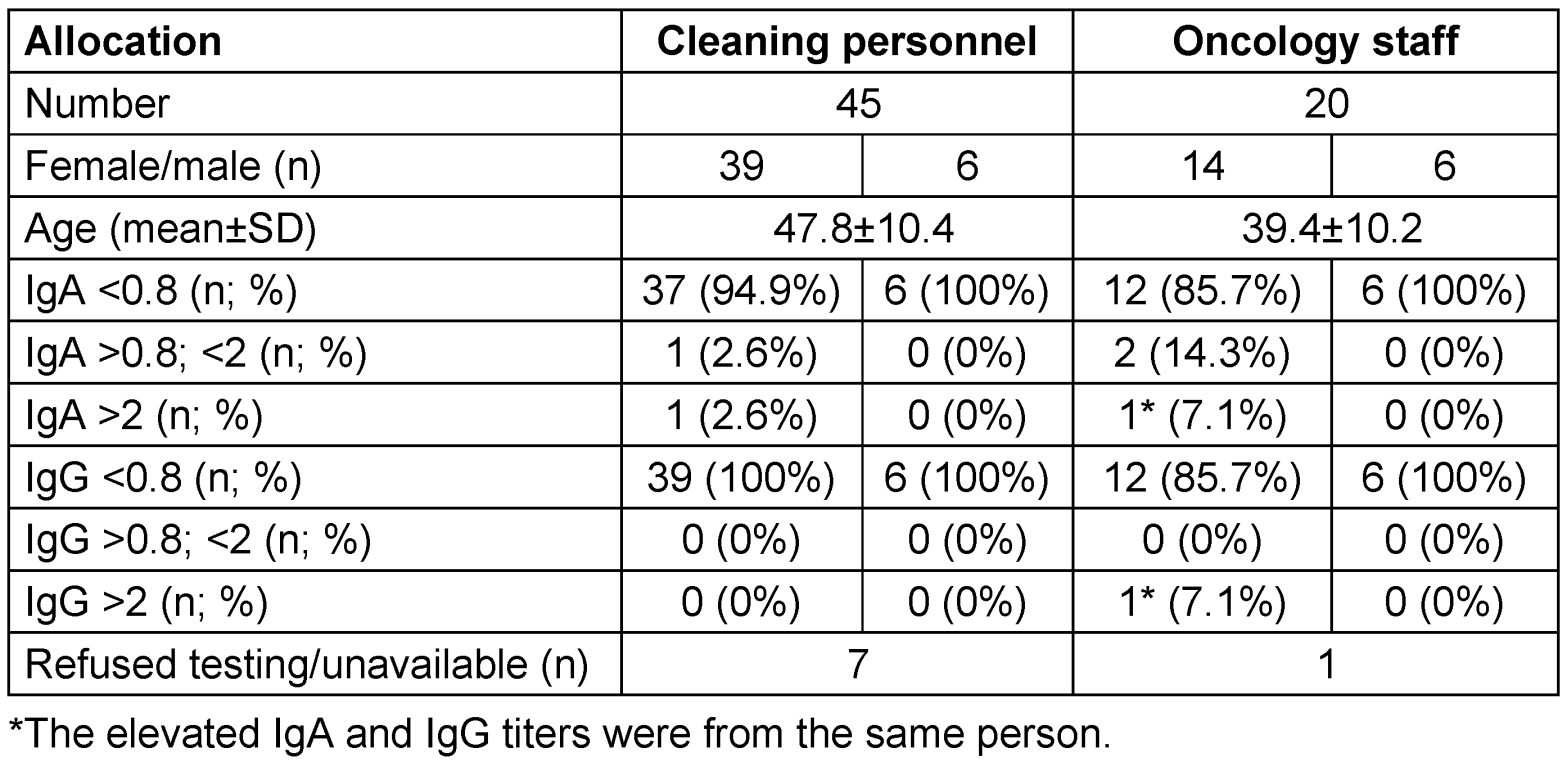
Anthropometric parameters and antibody titers in the cleaning personnel and the members of the oncological ward differentiated by sex
